# Supplementary material for: Chromothripsis during telomere crisis is independent of NHEJ, and consistent with a replicative origin
Source: Genome Res. 2019 May;29(5):737–49. doi: 10.1101/gr.240705.118 (PMC6499312; doi:10.1101/gr.240705.118)
Supplement: Supplemental Material [file supp_gr.240705.118_Supplemental_file_1.zip › contigs/annotated_contigs/DB93/contig.2.DB93_length_177_mean_cov_19.3050847458.docx]

**DB93_length_177_mean_cov_19.3050847458**

```
CCCCTTTAGGCAGAGCTTAGACCAGAGTTAGG|TCACCTGGGTGATCAGTGCAGGGATATGTCACAA|AGCACCCTGTAGGCAGATCCT
 >chrY:13475774-13475840 + E=1e-19 p=0e+00 >chr18:108358-108503 - E=2e-61
AGAGAAGAGTTATATCACCTGGGTGATCAGTGCAGAGGTAGGTCACAATCCCCCTGTAGGCAGAGCCTATACAAGAGTTATATCACCTG

G
```
